# Antimicrobials in small-scale urban pig farming in a lower middle-income country – arbitrary use and high resistance levels

**DOI:** 10.1186/s13756-018-0328-y

**Published:** 2018-03-07

**Authors:** G. Ström, S. Boqvist, A. Albihn, L.-L. Fernström, A. Andersson Djurfeldt, S. Sokerya, T. Sothyra, U. Magnusson

**Affiliations:** 1 Department of Clinical Sciences, Swedish University of Agricultural Sciences, Uppsala, Sweden; 2 Department of Biomedical Sciences and Veterinary Public Health, Swedish University of Agricultural Sciences, Uppsala, Sweden; 3 Department of Chemistry, Environment and Feed Hygiene, National Veterinary Institute, Uppsala, Sweden; 4 Department of Human Geography, Lund University, Lund, Sweden; 5 Centre for Livestock and Agriculture Development, Phnom Penh, Cambodia; 6 National Animal Health and Production Research Institute, Phnom Penh, Cambodia

**Keywords:** Antimicrobial use, Antimicrobial resistance, Pig production, Cambodia

## Abstract

**Background:**

Administration of antimicrobials to food-producing animals is regarded as a major contributor to the overall emergence of resistance in bacteria worldwide. However, few data are available on global antimicrobial use and resistance (AMR) in livestock, especially from low- and middle-income countries.

**Methods:**

We conducted a structured survey of 91 small-scale pig farms in the urban and peri-urban areas of Phnom Penh, Cambodia, to assess the farmers’ knowledge, attitudes and practices related to antimicrobial use in their pig production. Commensal *Escherichia coli* was isolated from three healthy pigs from each farm (*n* = 261) and susceptibility testing was performed against 14 antimicrobials, using broth microdilution. Univariable logistic regression and generalized linear mixed models were used to investigate potential associations between farm characteristics, management factors and resistance to different types of antimicrobials.

**Results:**

We found a widespread and arbitrary use of antimicrobials, often based on the farmer’s own judgment. Around 66% of the farmers reported frequently self-adjusting treatment duration and dosage, and 45% had not heard about the term ‘antimicrobial resistance’. The antimicrobials most commonly mentioned or kept by the farmers were amoxicillin, tylosin, gentamicin and colistin. Around 37% used a feed concentrate that contained antimicrobials, while antimicrobials for humans were used as a last-line treatment by 10% of the farmers. Commensal *E. coli* exhibited high prevalence of resistance to several antimicrobials considered to be of critical importance for human medicine, including ampicillin, ciprofloxacin and colistin, and multidrug-resistance was found in 79% of the samples. Higher prevalence of resistance was observed on farms that administered prophylactic antimicrobials and on farms that treated the entire group or herd in the event of disease.

**Conclusion:**

The widespread and arbitrary use of antimicrobials in pig farming in Cambodia is highly worrisome. Overall, farmers had a low awareness of the risks and consequences related to antimicrobial use and AMR. The results presented in this study confirm the hypothesis that non-rational use of antimicrobials results in higher prevalence of AMR and highlight the need for professional animal health systems that involve medically rational use of antimicrobials in emerging economies such as Cambodia.

**Electronic supplementary material:**

The online version of this article (10.1186/s13756-018-0328-y) contains supplementary material, which is available to authorized users.

## Background

Antimicrobial resistance (AMR) is a major global health concern and our decreased ability within human and veterinary medicine to cure resistant infections will have serious repercussions for future treatment and prevention of infections in humans and for the productivity of livestock [1–3]. The emergence of AMR is believed to be partly caused by the inappropriate use within the livestock sector, where antimicrobials are widely administered to animals for prevention and control of diseases and, more controversially, for growth promotion purposes [4–6]. Although data from several high-income countries indicate extensive antimicrobial use and AMR in livestock in these countries [7, 8], data from most low- and middle-income countries are scarce. Historically, the primary problem in some low- and middle-income countries has been the lack of antimicrobial drugs [9, 10]. Recently, however, the access to antimicrobials has generally increased in urban areas in these countries, with a consequent rise in the inappropriate usage of such drugs in cities and towns [11]. Overall, the use of antimicrobials in animal production is expected to continue to increase globally due to growing demand for animal-source foods, particularly in emerging economies [12]. Facilitated by the ongoing globalization of trade and travel, AMR is rapidly spread throughout the world [13].

Southeast Asia, a region with a high burden of infectious diseases [14] and a dense population of both humans and animals, is considered to be a hotspot for the development of AMR [15]. This partly stems from the expanding pig and poultry sectors [16] and partly from the widespread availability of antimicrobials and an often weak regulatory framework governing their use in human and veterinary medicine. Weak regulations enable inappropriate usage of antimicrobials which, in the livestock sector, often manifests as use without proper diagnosis, poor adherence to dosage and treatment duration, and use of falsified pharmaceuticals, behaviors that may favor AMR emergence [17, 18].

Antimicrobials are presumed to be extensively used in Southeast Asia, but only a few published studies have investigated practices related to antimicrobial usage in the large and expanding pig sector in the region [19–22]. These studies indicate uncontrolled and arbitrary use of antimicrobials with little or no supervision by veterinarians. Concomitantly, other studies have reported high prevalence of AMR in bacteria isolated from pigs and poultry in Southeast Asia, with resistance levels as high as 98% to ampicillin [23], 96% to tetracycline [24], and 97% to gentamicin [25]. Notably, resistance levels have been reported to be higher in pigs than in other species [26, 27].

The present study was undertaken in urban and peri-urban areas of Cambodia, where availability of antimicrobials was assumed to be good and in a country with strong economic growth [28], where regulations governing the use of veterinary antimicrobials are under development, but not yet implemented. The aim was to obtain information on knowledge, attitudes and practices related to antimicrobial use in small-scale pig farming in Phnom Penh, and data on phenotypic AMR in the indicator bacteria *Escherichia coli* isolated from pigs kept by the farming households. This information could assist in the development of strategies for more medically rational use of antimicrobials in the pig sector, not only in Cambodia but also elsewhere in the Global South.

## Methods

### Study area and study population

This cross-sectional study was conducted in urban and peri-urban areas of Phnom Penh in January and February 2017. Only households keeping pigs under family farm conditions (FAO, 2014) were targeted for the study, and thus larger commercial farms were excluded. Cambodian households have a long tradition of raising animals in their backyards for both subsistence and commercial purposes, with around 80% of the pigs in Cambodia still being raised in backyard systems [29].

In recruitment of farms, a list of pig-keeping households (family farms) in the province of Phnom Penh (*n* = 267) was available from a previous study [30]. In that study, snowball sampling [31] had been used with the ambition to locate all family farms keeping pigs in Phnom Penh. For the present study, all farms on that list were visited and included if they still kept pigs and if the farmers were at home and willing to participate in the study. However, due to failing profitability from pig production, a large proportion of the farms on the list had stopped keeping pigs since the previous study, and thus only 81 farms could be included from the previous study. However, ten farms that had started with pig production since the previous study were found during the fieldwork and were included in the present study.

### Study procedure

A semi-structured questionnaire, with questions on farm characteristics, pig husbandry and routines for antimicrobial (i.e. antibacterial) use, was administered to the person responsible for treating sick pigs at the farm. The questionnaire, which took around 20 min to complete, was written in English and interviews were carried out in Khmer with the assistance of the same interpreter throughout the study. The interpreter, an employee at the Centre for Livestock and Agriculture Development (CelAgrid), had extensive knowledge of Cambodian livestock production and was familiar with the different medicines and treatment regimens commonly used in livestock production in Cambodia. In order to determine whether the drugs referred to by respondents contained antimicrobials, probing questions were used together with observations of any medicines, premixes or feed concentrates present at the farm.

The questionnaire is provided as supplementary material [see Additional file 1].

### Collection of fecal samples

Fecal samples were collected from three randomly chosen healthy pigs at each farm, using sterile cotton swabs. At two of the 91 farms, no family member was at home when the team arrived for sampling and at some farms only one or two pigs were available for sampling, resulting in a total of 261 samples from 89 farms. Samples were collected either from the rectum or from fresh feces on the ground, if the pig defecated during sample collection. The swabs were placed in sterile plastic tubes containing Amies medium (Amies PS Viscose, Sarstedt), and stored and transported on ice to the National Animal Health and Production Research Institute (NAHPRI) in Phnom Penh within 8 h. Samples were stored at 2–8 °C until analysis, which was performed within 48 h after sampling.

### Isolation of *E. coli*

Fecal sampled were cultured on MacConkey agar at 44 °C overnight. Presumptive *E. coli* isolates were sub-cultured on Tryptone Soya Agar (TSA) and incubated at 37 °C overnight. Selected isolates were tested for production of tryptophanase (indole). One positive isolate was selected from each fecal sample, and bacterial material was transferred to cryogenic vials containing Luria Broth (LB) and 20% glycerol, and stored at − 70 °C before being transported to the Swedish University of Agricultural Sciences (SLU), Uppsala, Sweden.

### Antimicrobial susceptibility testing

Susceptibility testing was performed at SLU by broth microdilution, using the growth method for inoculum preparation, according to the standards described by the Clinical and Laboratory Standards Institute [32]. Prior to analysis, all isolates were confirmed as *E. coli* isolates by matrix-assisted laser-desorption/ionization time-of-flight mass spectrometry (MALDI-TOF MS).

Microdilution susceptibility panels (Sensititre™ EUVSEC, Thermo Scientific) were used to determine susceptibility of the bacteria isolates to 14 antimicrobials (ampicillin, azithromycin, cefotaxime, ceftazidime, chloramphenicol, ciprofloxacin, colistin, gentamicin, meropenem, nalidixid acid, sulfamethoxazole, tetracycline, tigecycline, trimethoprim). *Escherichia coli* CCUG 17620 was used as a quality control strain.

The minimum inhibitory concentration (MIC) for each antimicrobial was visually determined and epidemiological cut-off values (ECOFFs), defined by the European Committee on Antimicrobial Susceptibility Testing [33], were used to differentiate between wild-type and non-wild-type strains of the bacteria isolates, henceforth referred to as susceptible and resistant, respectively. Multidrug-resistance (MDR) was defined as isolates resistant to at least three different categories of the antimicrobials tested [34].

### Data management and statistical analysis

Data from the questionnaire responses were transcribed into Epi Info™ 7 (CDC, Atlanta, GA) following the interviews, and were exported on to Microsoft Excel 2010 spreadsheets after the fieldwork was finished.

Statistical analyses were conducted in SAS software 9.4 (SAS Institute Inc., Cary, NC). Descriptive statistics were computed to define farm characteristics and to determine knowledge, attitudes and practices among respondents regarding use of antimicrobials. Distributions for continuous variables were tested for normality using the Shapiro-Wilks test. Univariable logistic regression and Chi-square tests were used to examine possible associations between farm size, education level and management factors, such as routines related to antimicrobial use. To investigate associations between farm characteristics, management factors, age group of pigs and the prevalence of resistance to different types of antimicrobials, generalized linear mixed models were used where farm was included as a random effect, to account for clustering. The confounding variable ‘age group of pigs’ was included in all models as a fixed effect. The statistical significance level was defined as a two-tailed *P*-value ≤0.05 for all models. For the associations with AMR prevalence, however, all *P*-values < 0.1 are presented.

## Results

### Farm characteristics

Among the 91 farms included in the study, 29 farms (32%) also kept cattle and 64 farms (70%) kept poultry, besides keeping pigs. The number of pigs present in at the farm at the time of the visits ranged from 2 to 228 with a median of 20 pigs (5th and 95th percentiles: 7 and 81 pigs). The farm with 228 pigs was a farm that produced piglets to sell on to other producers, which explains its much higher number of pigs compared with the other farms.

Male household heads had in general attained a higher level of education than female household heads (*P* < 0.001). A high level of education was defined as commencing, but not necessarily completing, studies at upper secondary school, which was achieved by 31% of male and 9% of female farmers.

### Antimicrobial use

At 60 farms (66%), the male household head was responsible for treating sick animals while the female household head was responsible at 15 farms (16%) and the veterinarian or village animal health worker at nine farms (10%). At the remaining farms (8%), another household member (than the household heads) had the responsibility for treatment. The age of the person responsible for treating sick pigs (not including veterinarians or animal health workers) varied between 20 and 68 years, with a mean of 44 years. At 78 farms (86%) antimicrobials were routinely administered to the pigs to treat an existing disease, as a prophylactic for piglets or sows after farrowing, or as a feed additive commonly included in the feed concentrate. At nine farms (10%), however, it could not be determined with certainty that antimicrobials were used, as the respondent did not remember the names of the drugs used, and no drug packages were present. Nonetheless, all these farmers reported that they received drugs from veterinarians or pharmaceutical companies for treatment. Based on the respondents’ descriptions of disease symptoms and treatment regimens, we concluded that these farmers most likely administered antimicrobials to the pigs. Only four farmers (4%) reported that they did not use any antimicrobials, of which three farmers explained that their pigs had not been sick in a long time, while one farmer explained that they had stopped treatment with antimicrobials because their pigs died the last time they received antimicrobial treatment.

At least 70 different brands of antimicrobial drugs were used by the farmers. The antimicrobials most commonly mentioned or kept by the respondents were amoxicillin, tylosin, gentamicin and colistin (Table 1). Most respondents, however, could only name some of the antimicrobials that they used. Furthermore, the majority of piglet-producing farms gave the new-born piglets iron supplements, although only 24 respondents (26%) could specify or show the type of supplement used. Of these, eight farmers (33%) used iron supplements that contained antimicrobials, commonly gentamicin or colistin, in combination with tylosin, streptomycin or spectinomycin.

**Table 1:** Antimicrobials most commonly mentioned or kept by the farmers surveyed^a^

Antimicrobial	Number of farms
Amoxicillin^b^	56
Ampicillin^b^	21
Colistin^b^	27
Enrofloxacin^b^	19
Gentamicin^b^	29
Lincomycin	14
Oxytetracycline	17
Penicillin G^b^	15
Spectinomycin	6
Streptomycin^b^	9
Sulfonamides	16
Thiamphenicol	6
Trimethoprim	9
Tylosin^b^	38

^a^This list is not complete, as most farmers could only name a few of the antimicrobials that were used and it does not include potential antimicrobials in the feed concentrate

^b^Antimicrobial considered critically important according to WHO [48]

At least 14 farmers reported to routinely administer antimicrobials to the pigs as a preventive measure (Table 2). However, the antimicrobial content in the feed concentrate is not included in this figure and comprehensive information regarding the contents of the iron supplements administered could not be retrieved. At least 37% of the farms routinely used a concentrate that contained antimicrobials, although the types and concentrations of the antimicrobials were only specified on the concentrates used at three farms. It was commonly the concentrate for younger pigs (as advised by the manufacturer) that contained antimicrobials.

**Table 2:** Practices related to antimicrobial use on the farms that presumably used antimicrobials (*n* = 87)

	Category	*n*	%
How do you administer antimicrobials to the pigs?	Injections when sick	87	100
	In feed/orally when sick	18	21
	In water when sick	1	1
	To sows after farrowing (n=58^a^)	5	9
	In feed routinely	8	9
If only some pigs are sick, to which pigs do you administer antimicrobials? (*n* = 86)	Only the sick pigs	65	76
	All pigs^b^	21	24
Do you administer antimicrobials as a prophylaxis?	Yes	14	16
	No	72	83
	Unsure^c^	1	1
Does the feed concentrate contain antimicrobials? (n = 91^d^)	Yes	34	37
	No	8	9
	Don’t know	42	46
	Don’t use concentrates	7	8
Do you sometimes give human medicines that contain antimicrobials to the animals?	Yes	9	10
	No	77	89
	Don’t know	1	1
Do you sometimes end treatment prematurely if the animal gets better?	Yes	57	66
	No	30	34
What do you do with antimicrobials that are left (and have expired)? (*n* = 84)	Throw away to pond/environment	36	43
	Bury	20	24
	Burn	3	4
	Take back to veterinarian/animal health worker	8	10
	Throw to the person collecting waste	11	13
	Keep at home	4	5
	Don’t know	2	2
Do you have a withdrawal period (according to instructions) between antimicrobial treatment and slaughter/trader collecting animals? (*n* = 81)	Yes	8	10
	No	38	47
	Don’t know	1	1
	Never been sick around time of slaughter	32	40
	Other	2	2

^a^Calculated based on number of farms that kept sows

^b^This category does not necessarily includes sows, as some farmers only used traditional medicines to treat sows

^c^Some substance was added to the feed routinely but the respondent did not remember the name

^d^Calculated based on all 91 farms in the study

The respondents reported that they commonly received antimicrobials from the veterinarian or village animal health worker. Most veterinarians either operated a pharmaceutical store or were employed by a pharmaceutical company. Respondents described unrestricted access to antimicrobials, and professional prior diagnosis of the animals seemed to be rarely practiced. Many respondents explained that they just went to the store and described the disease symptoms to acquire antimicrobials from the veterinarian. Other respondents claimed that they did not necessarily need veterinary advice, as they could decide which type of antimicrobial and dosage to administer based on previous experiences. In general, treatment based on experience was a common practice among the farmers. On at least 60 farms (66%), the respondent reported frequently deviating from the instructions provided by veterinarians or written on product labels, by adjusting both dosage and duration of treatment based on the severity of the disease and whether the animals recovered quickly or not. Respondents that reported this behavior were also more likely to use human antimicrobials on their pigs (*P* = 0.056). Furthermore, almost half of the respondents (47%) reported that they did not adhere to the withdrawal period stated on drug packaging or prescribed by the veterinarian, and pigs were often sold for slaughter during or directly after antimicrobial treatment. None of the behaviors described above was associated with the level of education of the household head.

### Knowledge and attitudes about antimicrobials and AMR

Only one respondent (a former animal health worker) explained that he knew what antimicrobials were and their mode of action. For the other respondents, their knowledge was limited to recognizing the names of some antimicrobials they frequently used. In general, all respondents (99%) were of the opinion that antimicrobials were necessary in order to keep their animals healthy (Table 3). All respondents save two considered antimicrobials to be easily accessible. It should be noted that these two respondents stated either that antimicrobials were often sold out when they arrived at the store, or that it was difficult to find ‘high quality antimicrobials’ from Europe. The latter was mentioned by other respondents too and some expressed concerns that locally produced antimicrobials (commonly imported from Vietnam) were not as efficient as antimicrobials imported from European countries.

**Table 3:** Knowledge and attitudes about antimicrobials and antimicrobial use among respondents (*n* = 91)

	Category	*n*	%
Is it important to give antimicrobials to animals?	Yes	82	90
	No	4	4
	Don’t know	5	5
Are antimicrobials needed to keep animals healthy?	Yes	90	99
	No	0	0
	Don’t know	1	1
Will the use of antimicrobials result in better growth of animals? (*n* = 90)	Yes	30	33
	No	58	64
	Don’t know	2	2
Do you think it is easy to get access to antimicrobials?	Yes	89	98
	No	2	2
Do you consider antimicrobials to be cheap?	Yes	5	5
	No	55	60
	It’s acceptable	29	32
	Don’t know	2	2
Do you think giving antimicrobials to animals may result in any negative consequences?	Yes	49	54
	No	3	3
	Don’t know	39	43
Have you ever heard of ‘antimicrobial resistance’?	Yes	50	55
	No	41	45
Do you feel you have received enough information on how antimicrobials should be used in animals?	Yes	37	41
	No	38	42
	Don’t know	16	18

Around half of the respondents (55%) stated that they had heard of the term ‘antimicrobial resistance’. This was more likely among respondents who had attained a higher level of education (78% vs. 49%; *P* = 0.036; Odds ratio (OR) 3.6; 95% Confidence Interval (CI) 1.1–12.0) and was more often reported by male respondents (74% vs. 39%; *P* < 0.001; OR 4.4; CI 1.8–10.9), and by farms where the respondent was the person responsible for treating sick pigs (65% vs. 41%; *P* = 0.021; OR 2.7; CI 1.6–6.4). Some respondents elaborated on this question and gave examples from their own experience of what they believed to be AMR (Table 4).

**Table 4:** Statements by respondents regarding their experiences and reflections on AMR

*“When we use too much of one type of antimicrobial and the animal does not recover, it may be because of resistance”*
Male, 52 years, higher education
*“Sometimes after we use the antimicrobials, the animals get more sick and do not recover”*
Female, 36 years, lower education
*“If we use only one kind of medicine for a long time and then the animal recovers, the next time it [the medicine] might not work”*
Male, 35 years, higher education
*“When I use this one [points at antimicrobial] the pigs will no longer recover”*
Male, 35 years, lower education

Although many respondents had heard about AMR, when asked to state some possible negative consequences from the use of antimicrobials none mentioned this as a potential problem. Still, respondents expressed some concerns about other possible adverse effects that could arise from antimicrobial use, for example the risk of antimicrobial residues being present in the meat and potential negative effects on the animals when they received too much antimicrobials (Table 5).

**Table 5:** Statements by respondents regarding potential negative effects of antimicrobial use

*“If too much medicine, there may be medicine in the meat and it will taste different”*
Female, 35 years, lower education
*“If too much the pig will grow slower and the cycle will take longer time. Maybe it will affect the consumer if the animal gets too much antimicrobials. The meat will be yellow.”*
Female, 51 years, lower education
*“When a consumer eats an animal that has received a lot of antimicrobials it may have an effect on human health.”*
Male, 48 years, lower education
*“If the people that use the medicine don’t know how to use it, the medicine will remain in the meat. [It] may affect the people who eat the meat.”*
Female, 36 years, lower education

### Antimicrobial susceptibility in *E. coli*

**Fig. 1:**
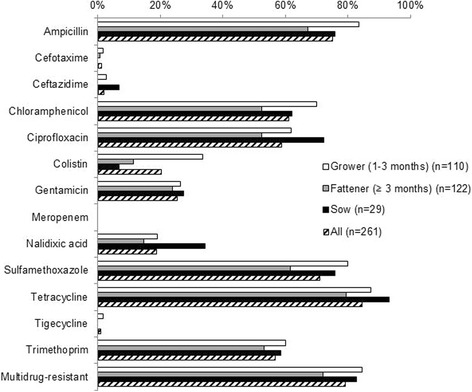
Prevalence of antimicrobial resistance of commensal *E. coli* isolated from growers, fatteners and sows

*Escherichia coli* bacteria were successfully isolated from all 261 samples, of which 110 were obtained from growers (1–3 months old), 122 were obtained from fatteners (over 3 months old), and 29 were obtained from sows. Overall, bacteria isolates were resistant to a median of five of the 13 antimicrobials tested (no ECOFF was available for azithromycin). Twenty-one isolates (8%) were susceptible to all antimicrobials, whereas 31 isolates (12%) showed resistance to eight or more antimicrobials. Among all isolates tested, resistance levels were as follows: ampicillin (AMP) 75%; cefotaxime (CTX) 1%; ceftazidime (CAZ) 2%; chloramphenicol (CHL) 61%; ciprofloxacin (CIP) 59%; colistin (COL) 20%; gentamicin (GEN) 25%; meropenem (MER) 0%; nalidixic acid (NAL) 19%; sulfamethoxazole (SMX) 71%; tetracycline (TET) 84%; tigecycline (TIG) 1%; and trimethoprim (TMP) 57%. Multidrug-resistance (MDR) was found in 79% of all isolates. Isolates from growers showed higher prevalence of resistance to all antimicrobials compared with isolates from fatteners (Fig. 1), although this difference was only significant for AMP, CHL, COL and SMX, and for MDR. Furthermore, isolates from sows showed significantly higher prevalence of resistance to CIP and NAL, compared with isolates from fatteners. Resistance prevalence and MIC distributions are presented for growers in Table 6, for fatteners in Table 7 and for sows in Table 8.

**Table 6:**
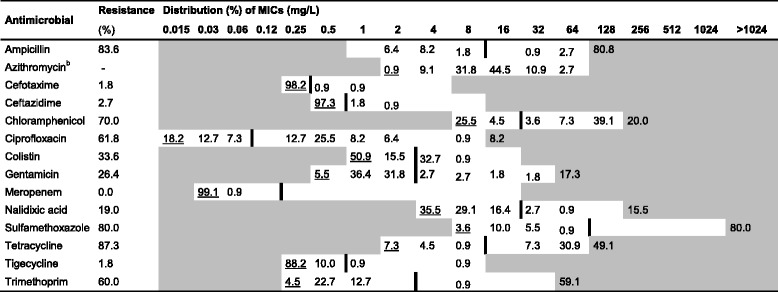
Resistance and distribution of Minimum Inhibitory Concentrations (MIC) of antimicrobials for *E. coli* from growers^a^ (*n* = 110)

White fields represent range of dilutions tested for each antimicrobial. MICs higher than the highest concentration tested are given as the concentration closest above the range. MICs equal to or lower than the lowest concentration tested are underlined. The epidemiological cut-off value (ECOFF) [33] for each antimicrobial is presented as a vertical line

^a^Pigs aged between 1 and 3 months

^b^No ECOFF was available for azithromycin

**Table 7:**
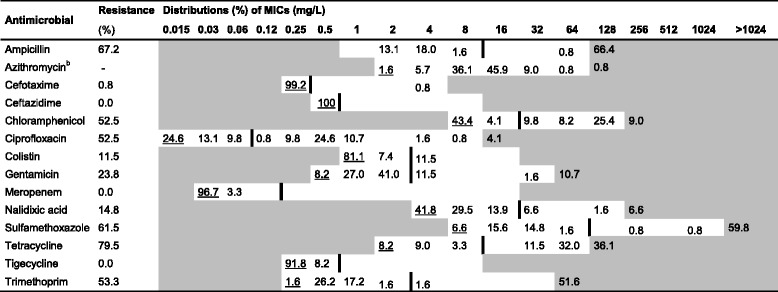
Resistance and distribution of Minimum Inhibitory Concentrations (MIC) of antimicrobials for *E. coli* from fatteners^a^ (*n* = 122)

White fields represent range of dilutions tested for each antimicrobial. MICs higher than the highest concentration tested are given as the concentration closest above the range. MICs equal to or lower than the lowest concentration tested are underlined. The epidemiological cut-off value (ECOFF) [33] for each antimicrobial is presented as a vertical line

^a^Pigs older than 3 months

^b^No ECOFF was available for azithromycin

**Table 8:**
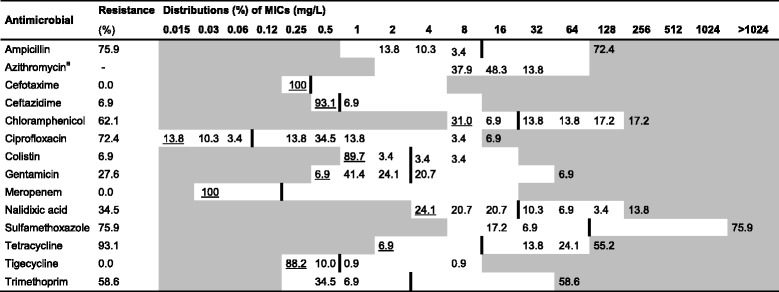
Resistance and distribution of Minimum Inhibitory Concentrations (MIC) of antimicrobials for E. coli from sows (*n* = 29)

White fields represent range of dilutions tested for each antimicrobial. MICs higher than the highest concentration tested are given as the concentration closest above the range. MICs equal to or lower than the lowest concentration tested are underlined. The epidemiological cut-off value (ECOFF) [33] for each antimicrobial is presented as a vertical line

^a^No ECOFF was available for azithromycin

### Antimicrobial use, farm characteristics and associations with AMR

The statistical analyses revealed some associations between farm characteristics, practices related to antimicrobial use and the prevalence of resistance to the antimicrobials tested. Age of the person responsible for antimicrobial treatment was positively related to lower prevalence of resistance to CIP (*P* = 0.043) and SMX (*P* = 0.021), and to MDR (*P* = 0.029). Isolates from pigs owned by farmers who had attained a higher level of education showed a higher prevalence of resistance to CIP (*P* = 0.007) and NAL (*P* = 0.053), and to MDR (*P* = 0.024). There was no correlation between age of the farmer and level of education. Higher prevalence of resistance was also found for NAL (*P* = 0.031), TMP (*P* = 0.004) and SMX (*P* = 0.023), and for MDR (*P* = 0.089) in isolates from farms where a veterinarian was responsible for antimicrobial treatment. Furthermore, isolates from farms where antimicrobials were administered preventatively showed higher prevalence of resistance to AMP (*P* = 0.081), CIP (*P* = 0.072), CHL (*P* = 0.056), SMX (*P* = 0.028) and TMP (*P* = 0.031), and farms that often treated the entire group of pigs instead of only the sick individuals had a higher prevalence of resistance to CIP (*P* = 0.021), COL (*P* = 0.048) and NAL (*P* = 0.012). Finally, farms that reported routinely using feed supplements that contained antimicrobials had a higher prevalence of resistance to AMP (*P* = 0.070), CIP (*P* = 0.076), CHL (*P* = 0.015), SMX (*P* = 0.031) and TMP (*P* = 0.016). For farms where it could be concluded that the concentrate included antimicrobials, the prevalence of resistance to CHL (*P* = 0.068) was higher.

## Discussion

In the present study, we found that the majority of the participating farms used antimicrobials in their pig production. Overall, knowledge regarding antimicrobial use appeared to be low among the respondents and few reported having received any training in veterinary practices. Antimicrobial use was mainly based on pig-keepers’ experiences and on drug sellers’ advices according to symptoms described by the owner, rather than being based on professional diagnostics. Similar practices have been reported in other studies conducted in the region [20, 21]. In the present study, we frequently found labels on the veterinary antimicrobials in languages other than Khmer, such as English, Vietnamese or Spanish. Additionally, labelling on concentrate packaging and feed pre-mixes did not always specify the type and concentration of the antimicrobial content. This prevents farmers and retailers from making informed decisions on which drugs to choose and may contribute to inappropriate use of antimicrobials in livestock farming in Cambodia.

In agreement with other studies in Southeast Asia [23, 24, 27] we found a high prevalence of resistance to ampicillin, tetracycline and chloramphenicol, probably reflecting a long tradition of use in livestock farming in the region. The high prevalence of resistance to colistin, especially among growers, is a particular concern, as colistin is considered a last-resort antimicrobial for treatment of severe human infections and its use in livestock production may contribute to emerging resistance globally. The high prevalence of resistance detected in this study most likely results from the widespread use of colistin, either for treatment ofdiseases or as a prophylactic for piglets. We also found high prevalence of resistance to the quinolone ciprofloxacin, an antimicrobial not licensed for veterinary use. This is probably explained by cross-resistance with other veterinary quinolones, such as enrofloxacin [35], an antimicrobial commonly used by the farms in the present study. Notably, higher prevalence of AMR was found on farms that reported administering antimicrobials as a prophylactic and on farms that normally treated the entire group or herd in the event of disease. These results support the claim that non-rational use of antimicrobials contributes to increased prevalence of AMR [5].

We found that bacteria isolated from growers (1–3 months) exhibited significantly higher prevalence of resistance to several of the antimicrobials tested, including MDR, than bacteria from fatteners (older than 3 months). Higher prevalence of AMR in younger pigs has been reported in other cross-sectional studies [36, 37]. A longitudinal study by Nguyen et al. [19] presented similar results where the prevalence of resistance to ciprofloxacin and gentamicin, as well as MDR, in *E. coli* declined during the rearing process. The decreasing prevalence of AMR is suggested to be the result of reduced use of antimicrobials during the finishing phase of pig production and may also reflect the potential fitness cost of resistance in bacteria from the intestinal tract [38]. In the present study, however, isolates from sows exhibited significantly higher prevalence of resistance than isolates from fatteners for several of the antimicrobials tested. One explanation might be that sows (and also younger pigs) receive oral antimicrobials to a higher extent than older pigs, as oral administration of antimicrobials has been reported to increase AMR in commensal *E. coli* from pigs [39]. Animal age might thus be an important factor to consider when investigating prevalence of AMR in pigs.

Two thirds of the farmers reported frequently deviating from the instructions provided by veterinarians or those written on drug packaging. Common practices were to stop administering antimicrobials as soon as the animals started to recover, hence not completing the treatment, and to self-adjust the dosage based on the severity of the disease. Poor adherence to recommended instructions may increase the risk of AMR [17, 18] and has been reported to be a common problem in countries with non-prescription access to antimicrobials [40]. A particular concern is the use of human antimicrobials as a last-line treatment and the handling of expired antimicrobials, where almost half of the respondents stated that they commonly dumped the remaining pharmaceuticals in the environment once the expiry date had passed. This implies that discarded antimicrobials may be present locally at high concentrations in the environment, where they may contribute to resistance development [41, 42].

Improper and unregulated use of antimicrobials may pose a risk to public health if antimicrobial residues are present in animal products. Nearly half of the respondents in the present study reported that pigs were commonly sold during or directly after antimicrobial treatment, that is within the prescribed withdrawal time. If withdrawal times are not respected there is a risk of antimicrobial residues remaining in the meat at slaughter [43]. In many low- and middle-income countries, compliance with withdrawal times is not monitored and analysis of animal products is not routinely practiced. Studies in Vietnam, however, have found tetracycline [44] and sulfamethazine [45] residues in 5.5 and 8.8%, respectively, of pork meat sampled at local markets.

The prevalence of resistance to several antimicrobials, including MDR, was lower on farms operated by an older farmer, possibly as a consequence of more experienced farmers having better disease control practices, as previously suggested by Nhung et al. [27]. Notably, farms on which a veterinarian was responsible for antimicrobial treatment had a higher prevalence of resistance to several of the antimicrobials tested, including MDR. This is a concern, as veterinary supervision is a measure often put forward when discussing ways to improve antimicrobial treatment regimens. It is possible that these farmers relied on a veterinarian for treatment as a result of poor knowledge of animal health and veterinary practices, which in turn might result in deficient disease control. However, it is also important to consider the fact that veterinarians in most countries globally (including Cambodia) receive a considerable part of their income from the sale of pharmaceuticals, and therefore may be reluctant to reduce the use of antimicrobials in their practice [46]. It is also possible that these results reflect a need to strengthen veterinary education in Cambodia.

Male respondents more often stated that they had heard about antimicrobial resistance than female respondents. This could be a consequence of men generally attaining a higher level of education than women, or of men most commonly being responsible for treating sick pigs and therefore probably having more discussions with veterinarians. The latter suggestion is reinforced by the fact that previous awareness of antimicrobial resistance was more commonly reported by farms where the respondent was the person responsible for treating sick pigs. Although our aim was to interview the person responsible, this was not possible on all farms. If we had omitted those farms, we would probably have ended up with far fewer farms in our study.

When performing surveys that rely on self-reported practices, there is always the risk of recall bias or of respondents providing answers that they think are ‘correct’, but which may not correspond to actual practices. In the present study, many of the respondents did not remember the names of the drugs they used and they did not keep any treatment records. Thus we were unable to determine whether higher levels of AMR were the result of higher consumption of certain antimicrobials or to establish any associations between antimicrobial use and explanatory factors, such as farm size or education level.

## Conclusions

The emergence of AMR is a truly global issue as it spreads easily between countries, and excessive and inappropriate use of antimicrobials should thus be mitigated everywhere. In the present study, we found that antimicrobial use in urban and peri-urban pig farming in Cambodia was commonly only based on farmers’ experiences or on drug sellers’ advices based on descriptions of symptoms, and there was low awareness of the risks and consequences of antimicrobial resistance among the respondents. Commensal *E. coli* from pigs showed high prevalence of AMR to several antimicrobials considered to be of critical importance for human medicine, including ampicillin, ciprofloxacin and colistin, and multidrug-resistance was found in four out of five samples. Higher prevalence of resistance was found on farms that administered antimicrobials as a prophylactic and on farms that treated the entire group or herd in the event of disease. These results confirm the hypothesis that non-rational use of antimicrobials results in higher prevalence of AMR and highlight the need for professional animal health systems that involve medically rational use of antimicrobials in emerging economies such as Cambodia.

## Additional file

Additional file 1: The questionnaire. (DOCX 225 kb)

## Abbreviations

AMP: Ampicillin
AMR: Antimicrobial resistance
CAZ: Ceftazidime
CHL: Chloramphenicol
CIP: Ciprofloxacin
CLSI: Clinical and Laboratory Standards Institute
COL: Colistin
CTX: Cefotaxime
ECOFF: Epidemiological cut-off value
EFSA: European Food Safety Authority
EUCAST: European Committee on Antimicrobial Susceptibility Testing
GEN: Gentamicin
MDR: Multidrug-resistance
MER: Meropenem
MIC: Minimum Inhibitory Concentration
NAHPRI: National Animal Health and Production Research Institute
NAL: Nalidixic acid
Sida: Swedish International Development Cooperation Agency
SLU: Swedish University of Agricultural Sciences
SMX: Sulfamethoxazole
TET: Tetracycline
TIG: Tigecycline
TMP: Trimethoprim
TSA: Tryptone Soya Agar
VR: Swedish Research council
WHO: World Health Organization
