# Supplementary material for: Antimicrobials in small-scale urban pig farming in a lower middle-income country – arbitrary use and high resistance levels
Source: Antimicrob Resist Infect Control. 2018 Mar 7;7:35. doi: 10.1186/s13756-018-0328-y (PMC5842516; doi:10.1186/s13756-018-0328-y)
Supplement: Supplementary file 1 — The questionnaire. (DOCX 225 kb) [file 13756_2018_328_MOESM1_ESM.docx]

# Knowledge, Attitude, Practice (KAP) Study - Antibiotic use among livestock-keepers in Phnom Penh

## Identification

| 1.1. ID |  | |
| --- | --- | --- |
| 1.2. Date of Survey  (DD/MM/YYYY) | /            /    2017 | |
| 1.3. Khan  1.4 Region |  | |
| 1.5 GPS Coordinates | Lat. (N): | Long. (E): |

## Demographics

2.1 Who in the household is responsible for treating/handling sick animals?

- Male HH head
- Female HH head
- Other male HH member
- Other female HH member
- Other, specify                                                                
  *(Ask to speak to that person)*

2.2 Age of the person responsible for treating/handling sick animals:                    years

2.3 What is the highest grade of formal education completed for…?

You:                           Your spouse:                          *(“you”refers to the person responsible)*

| **Education level** | |
| --- | --- |
| 0 = No formal education  1 = Primary education  2 = Completed Lower Secondary school (7-9^th^ grade)  3 = Some Lower Secondary school  4 = Completed Upper Secondary school (10-12^th^ grade)  5 = Some Upper Secondary school | 6 = Vocational training (specify no of yrs.)  7 = Tertiary training specify yrs.  8 = University degree (undergraduate)  9 = Some University training  10 = University degree (postgraduate)  11 = Other, specify |

2.4 Have you received any extension services or training about pig production?

- Yes No Don’t know

2.5 If yes, please specify:

## Livestock production

3.1 Indicate how many animals you keep currently:

**Pigs Cattle Poultry**

**
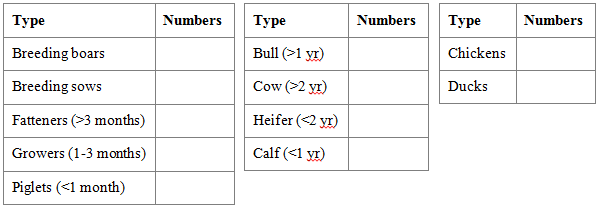
**

3.2 What type of pig production do you have?

*[FA = Fatteners; FF = Farrow-to-finish; P = Piglet; FFF = Combination FA and FF; FFP = Combination FF and P]*3.3 Do you buy pigs regularly? Yes No *Comments:*

3.4 If yes, from where/whom do you buy the pigs?

3.5 How many pigs have been born or bought in the past 12 months?

*Born*:            piglets *Bought*:            piglets            growers             fatteners             Other

3.6 For how long have you been keeping pigs?

3.7 Did you or any other member in your household have any previous experience in pig keeping, before you started? Yes No

3.8 If yes, what kind of experience?

## Antibiotic use

4.1 What type of medicines do you use for the animals? [9]=Don’t know

*Ask to see the “medicine cabinet” (take photos). Fill in details on last page.
Make sure the substances referred to really contain/are antibiotics.*

4.2 Do any of the medicines you use contain antibiotics? Yes No Don’t know

4.3 Do you give any other substances that contain antibiotics to the animals? For example feed additives, supplements etc (excl. concentrate and iron)? Yes No Don’t know
*Ask to see the additives/supplements (take photos). Fill in details on last page.*

4.4 Does the feed concentrate contain any antibiotics?

[1]=Yes [0]=No [9]=Don’t know [-]=Do not use concentrate

Pigs                   Cattle                    Chickens/ducks

*Ask to see the concentrates (take photos). Fill in details on last page.*

4.5 If you give any substances containing antibiotics to the animals, to which animal species?

Pig Cattle Chickens/ducks Other, specify

4.6 If the respondent does not use antibiotics to the animals, ask why not. *(Maybe used it before?)*

**If no antibiotics are used, continue to section 5.**

4.7 Can you explain the reason why you give antibiotics to the animals?

4.8 When, or how long ago, did you start to use antibiotics for the animals?

4.9 Did someone advise you to start with it? Yes No

4.10 If yes, who?

4.11 If no, how come you decided to start to give antibiotics?

4.12 Do you know what (names of) antibiotics you use? [1]=Yes [0]=No [9]=Know some

Pigs                    Cattle                   Chickens/ducks

4.13 Specify the names of the antibiotics you use and explain how and when they are used (**P**reventive, **T**reatment, **G**rowth). *Use this question to help answer Q 4.14-4.20.*

Pig:

Cattle:

Chickens/ducks:

4.14 How do you administer antibiotics to **pigs**?

- Iron for piglets Contain antibiotics? Yes No Don’t know
- To the feed regularly (excl. conc.) When/How often?
- To the water regularly When/How often?
- Injections regularly When/How often?
- To the feed when they are sick
- To the water when they are sick
- Injections when they are sick

4.15 To which categories of pigs do you give antibiotics regularly?

- All pigs *(still indicate which type of pigs below)*
- Gestation sows Piglets
- Nursing sows Growers
- Boars Fatteners

4.16 To which categories of pigs do you give antibiotics when sick?

- All pigs *(still indicate which type of pigs below)*
- Gestation sows Piglets
- Nursing sows Growers
- Boars Fatteners

4.17 If only some pigs in a pen are sick, to which animals do you give antibiotics?

To all pigs (in the pen) Only to sick pigs

4.18 How do you administer antibiotics to **cattle**?

- To the feed regularly (excl. conc.) When/How often?
- To the water regularly When/How often?
- Injections regularly When/How often?
- To the feed when they are sick
- To the water when they are sick
- Injections when they are sick

4.19 How do you administer antibiotics to **chickens/ducks**?

- To the feed regularly (excl. conc.) When/How often?
- To the water regularly When/How often?
- Injections regularly When/How often?
- To the feed when they are sick
- To the water when they are sick
- Injections when they are sick

4.20 If only some chickens/ducks are sick, to which animals do you give antibiotics?

To all chickens/ducks Only to sick chickens/ducks

4.21 How do you get access to antibiotics?

Veterinarian Local store/pharmacy Open market Other, specify

4.22 Do you get a prescription or instructions when you buy antibiotics?

Yes, mostly Sometimes No, never/very rarely Other, specify

4.23 If yes or sometimes, from where/by whom?

4.24 Who decides about when and the amount of antibiotics to give to the animals?

Veterinarian Respondent Household head  *(if other than respondent)*
 Other person, specify

4.25 If or when you don’t get any instructions, how do you decide which antibiotic/supplement to
 use and how to use it?

4.26 Have you or any other household member received any training in how to use antibiotics to the animals? Yes No

4.27 If yes, what kind of training?

4.28 Do you sometimes give more or less antibiotics than recommended? Yes No

4.29 If yes, how?

Lower dose Shorter treatment duration Other, specify

Higher dose Longer treatment duration

4.30 If yes, why?

4.31 Do you ever self-combine different antibiotics? Yes No Don’t know
 Only if vet/AHW advice it

4.32 If yes, how/why?

4.33 Do you sometimes give human medicines that contain antibiotics to the animals?
 Yes No
4.34 Do you sometimes stop giving antibiotics if the animal that is treated gets better during
 the course of treatment? Yes No Don’t know

4.35 Do you have a withdrawal period between administration of antibiotics and
 slaughter/trader collects animals? *(According to instructions)*
 Yes No Don’t know Not enough

4.36 What do you think would happen if you could not use antibiotics?

4.37 Do you always/commonly keep some antibiotics at home, in case an animal gets sick?

Yes No Don’t know

4.38 What do you do with antibiotics that are left after treatment?

## Knowledge and attitudes about use of antibiotics

5.1 Do you know what antibiotics are? Yes No

5.2 Please answer the following statements:

| a) | The use of antibiotics results in better growth of the animals | Yes No Don’t know |
| --- | --- | --- |
| b) | Antibiotics are needed to keep animals healthy | Yes No Don’t know |
| c) | It is easy to get access to antibiotics | Yes No Don’t know |
| d) | Antibiotics are cheap | Yes No Don’t know |

5.3 Do you think it is important to give antibiotics to the animals? Yes No

- 1. If yes, please explain why and when
  2. Do you think giving antibiotics to animals may result in any negative consequences?
      Yes No

5.6 If yes, what kind of consequences?

5.7 Have you ever heard about “antibiotic resistance”? Yes No

5.8 If yes, from whom/where?

5.9 Do you feel you have received enough information on how antibiotics should be used to animals? Yes No

5.10 If yes, from whom have you received information?

5.11 If no, what kind of information would you like to receive?

Other comments:
